# Efficacy of Biologics in Patients with Ulcerative Colitis Exhibiting Non-Response or Insufficient Response to Cytapheresis

**DOI:** 10.3390/jcm14186574

**Published:** 2025-09-18

**Authors:** Masahiro Iizuka, Takeshi Etou, Haruka Yorozu, Bunichiro Kato, Takeya Edagawa, Shiho Sagara, Hiro-O Matsushita, Kenjiro Yoshikawa

**Affiliations:** 1 Health Promotion Center, Japanese Red Cross Akita Hospital, 222-1 Kamikitade Saruta Aza Naeshirozawa, Akita City 010-1495, Japan; uzy1005uzy@gmail.com; 2 Department of Gastroenterology, Japanese Red Cross Akita Hospital, Akita City 010-1495, Japan; trhqm875@yahoo.co.jp (T.E.); h-yorozu.ij@jrc.or.jp (H.Y.); bunichiro_kato@akita-med.jrc.or.jp (B.K.); edage_0407@yahoo.co.jp (T.E.); mattsu@mui.biglobe.ne.jp (H.-O.M.); yoshikawaken_33@yahoo.co.jp (K.Y.)

**Keywords:** ulcerative colitis, cytapheresis, biologic therapy, granulocytes and monocyte adsorptive apheresis, anti-tumor necrosis factor-α, second-line therapy

## Abstract

**Background/Objectives**: Recent studies have investigated the effectiveness of second-line agents in patients with ulcerative colitis (UC) who have experienced failure with first-line biologics. Although cytapheresis (CAP) is an effective therapeutic strategy for refractory UC, the efficacy of second-line agents in patients with UC with previous CAP failure remains unexplored. Herein, we examined the efficacy of biologics in patients with refractory UC who experienced non-response or insufficient response to previous CAP. **Methods**: We retrospectively assessed the efficacy of biologics administered between January 2013 and June 2024 in patients with refractory UC who experienced non-response or insufficient response to previous CAP. Rates of clinical remission, steroid-free remission, and endoscopic improvement were also determined. Clinical remission was evaluated using Lichtiger’s clinical activity index. **Results**: Eighty-one patients with refractory UC underwent CAP, thirty of whom were eligible for study inclusion. The use of biologics was associated with clinical and steroid-free remission rates of 75.9% and 44.8%, respectively. Clinical and steroid-free remission rates of biologics did not differ significantly between patients with an insufficient response to previous CAP and those with non-response to previous CAP. There were no significant differences in clinical or steroid-free remission rates of biologics between patients with steroid-dependent and steroid-refractory UC. Endoscopic improvement was observed in 54.5% of patients. **Conclusions**: Despite the limited number of patients, biologic therapy was effective in patients with refractory UC who had experienced a non-response or insufficient response to previous CAP. Accordingly, biologics may be a useful second-line therapy for patients with refractory UC who have failed CAP.

## 1. Introduction

Ulcerative colitis (UC) is a chronic inflammatory bowel disease (IBD) of the colon characterized by a relapsing and remitting course [1,2]. Various factors, including genetic, environmental, and luminal factors, as well as mucosal immune dysregulation, reportedly contribute to the pathogenesis of UC [1]. Corticosteroids (CSs) are the first-line treatment for moderate-to-severe UC, resulting in complete remission in 54% of patients, partial remission in 30% of patients, and non-response in 16% of patients [3]. In spite of the effectiveness of CSs in UC, steroid dependence occurs in 17–22% of patients within 1 year of initial CS therapy, increasing to 38% within 2 years [3,4,5,6,7]. Steroid-dependent and steroid-refractory UC are collectively termed refractory UC.

Along with recent advancements in UC therapy, innovative treatments, including biologics, Janus kinase (JAK) inhibitors, sphingosphine 1-phosphate receptor modulators, and cytapheresis (CAP), have been developed for refractory UC [8,9]. However, appropriate therapeutic algorithms for these treatments remain inadequately established.

Biologic therapies, such as anti-tumor necrosis factor (TNF) inhibitors, anti-integrin antibodies, and anti-interleukin-12/23 monoclonal antibodies, have been shown to be highly effective in the induction of disease remission and long-term control in patients with UC [10]. However, approximately one-third of patients who initiate biologic therapy need to switch to second-line biologic therapy within one year owing to primary non-response or non-primary non-response [10,11]. Despite the availability of various biologic agents, physicians face challenges in the selection of drug and switching decisions. According to the guidelines, it is suggested that the mode of anti-TNF failure informs subsequent treatment decisions: for primary non-response, switching to a biologic with a different mechanism of action; for non-primary non-response, switching to a different anti-TNF [12,13,14]. In contrast, where economic considerations position anti-TNF therapies first in UC, several studies support the second-line use of vedolizumab (VDZ), regardless of the mode of failure of anti-TNF [11,12,15,16]. Zhang et al. recommended prioritizing infliximab (IFX) or VDZ as second-line biologic therapy for patients with UC who experienced first-line treatment failure [10]. Furthermore, Lee et al. showed that there is significant heterogeneity of treatment efficacy with different advanced therapies in inducing remission in patients with UC based on prior exposure to TNF antagonists, with plausible potentiation of JAK inhibitors and attenuation of lymphocyte trafficking inhibitors [17]. Two clinical studies have reported the efficacy of CAP as a second-line therapy in patients with UC who experienced biologic failure. Yamamoto et al. demonstrated that the clinical remission rate of granulocyte and monocyte adsorptive apheresis (GMA) in patients with UC was 31% in those who were exposed to biologics and 48% in those not exposed to biologics (*p* = 0.01) [18]. In a multivariate analysis, exposure to biologics was identified as a significant and independent factor affecting the clinical efficacy of GMA (*p* = 0.01). Dignass et al. reported that remission was achieved at week 12 of GMA in 40.3% of patients who experienced treatment failure with immunosuppressants and in 27.8% of patients who experienced treatment failure with anti-TNF-α therapy [19]. In addition, Fernández-Pérez et al. [20] analyzed the remission rate in patients with UC who were naive to immunomodulators (INMs) or biologics or had previously been exposed to any of these drugs. They stated that there was a clear tendency for naive patients to show a higher remission rate (50% at end of induction) than those exposed to INM or biologics (28% at end of induction). However, a statistically significant benefit was not found [20]. Despite the limited evidence, CAP tends to be less effective in patients with UC who were previously exposed to biologics or failed to respond to biologic therapy.

On the other hand, several studies showed that in a multivariate analysis, duration of UC [18], clinical or endoscopic severity [18,21,22], proctitis only [21], and exposure to corticosteroids [18], as well as exposure to biologics [18,21,22], were statistically significant predictors of achieving clinical remission in biologic or CAP therapy.

CAP is a non-pharmacological extracorporeal therapy developed as a treatment for UC [23,24,25,26,27,28,29]. CAP is performed using two methods: GMA, which uses cellulose acetate beads (Adacolumn, JIMRO Co., Ltd., Takasaki, Japan), and leukocytapheresis (LCAP), which utilizes polyethylene phthalate fibers (Cellsorba, Asahi Kasei Medical Co., Ltd., Tokyo, Japan) [23,30]. GMA selectively depletes elevated levels of granulocytes and monocytes from the patient’s circulation while sparing most of the lymphocytes [23]. LCAP exerts its anti-inflammatory effects by removing activated leukocytes or platelets from the peripheral blood via extracorporeal circulation [30]. CAP is known to be an effective therapeutic strategy for active refractory UC while exerting fewer adverse effects [23,24,25,26,27,28,29,31]. A meta-analysis revealed that GMA can effectively induce clinical remission in patients with UC compared with CS (odds ratio [OR], 2.23; 95% confidence interval [CI]: 1.38–3.60). Moreover, the rate of adverse events associated with apheresis was found to be significantly lower than that induced by CS (OR, 0.24; 95% CI: 0.15–0.37) [32]. Another study showed that compared with conventional pharmacotherapy that included CS, LCAP supplementation could offer substantial benefits in terms of promoting a rate of response (OR, 2.88, 95% CI: 1.60–5.18) and rate of remission (OR, 2.04, 95% CI: 1.36–3.07), along with notably greater steroid-sparing effects (OR, 10.49, 95% CI: 3.44–31.93) in patients with active moderate-to-severe UC [33]. Regarding the efficacy of CAP in patients with steroid-dependent UC, the rates of steroid-free remission and clinical remission ranged from 13% to 55% and 33.0% to 70%, respectively [19,30,31,34,35,36,37,38,39]. However, no clinical study has investigated the efficacy of second-line agents in patients with UC previously treated with CAP or those who failed to respond to CAP. Given that recent studies have investigated the effectiveness of second-line agents in patients with UC who experienced failure of first-line biologic therapy, it is crucial to optimize second-line therapies for patients with refractory UC after CAP treatment failure.

Accordingly, in the current study, we first assessed the efficacy of biologics as a second-line therapy for patients with refractory UC who experienced non-response or insufficient response to previous CAP. Furthermore, we discussed the optimal sequence of biologics and CAP therapies for patients with refractory UC.

## 2. Methods

### 2.1. Study Design

We retrospectively assessed the efficacy of biologics, including IFX, adalimumab (ADA), VDZ, and ustekinumab (UST)**,** administered mostly between January 2013 and June 2024 in patients with refractory UC showing no or insufficient response to previous CAP (GMA or LCAP) performed prior to biologic therapy. Basically, we defined CAP failure after 5 to 10 CAP sessions based on Lichtiger’s clinical activity index (CAI) [40]. In this study, we specifically defined patients whose CAI scores were not changed or not reduced to ≤4 after CAP therapy as those with a non-response to CAP. We also defined patients who responded to a previous CAP but did not achieve steroid-free clinical remission as those with an insufficient response to CAP. Patients with refractory UC included those with steroid-dependent and steroid-refractory patients. Steroid-dependent UC is defined as a disease that initially responds to steroid therapy, but symptom control cannot be maintained in the absence of steroids and requires low-dose steroid therapies for the symptom-free state to be maintained [2,19]. Additionally, steroid-refractory UC was defined as active UC characterized by the failure of the response to 0.75–1.5 mg/kg/day of prednisolone administered over at least 1 week [19,26]. This retrospective observational study was conducted in accordance with the Declaration of Helsinki and approved by the Institutional Review Board of the Japanese Red Cross Akita Hospital (approval no. 584, approval date 16 October 2023).

The primary endpoint was the rate of clinical remission after biologic therapy in patients with UC who experienced a non-response or insufficient response to previous CAP. The secondary endpoint was the rate of steroid-free remission after biologic treatment in the included patients with UC. We defined “steroid-free” as the point when both oral steroids and enemas, including steroids, were discontinued. However, suppositories containing small amounts of steroids are permitted as exceptions.

Further, we evaluated rates of maintenance of clinical remission and persistency of biologics at 104 weeks of therapy. In addition, we evaluated the relationship between the duration between CAP failure and biologic initiation and efficacy of biologics.

Endoscopic improvements after biologic therapy were also evaluated. Furthermore, the rates of clinical and steroid-free remission following biologic therapy were compared between the patients with insufficient response to previous CAP and the patients with non-response to previous CAP. We also compared the rates of clinical and steroid-free remission associated with biologic therapy in patients with steroid-dependent and steroid-refractory UC.

### 2.2. Evaluating the Efficacy of Biologics

Clinical efficacy was evaluated using Lichtiger’s clinical activity index (CAI) [40]. Clinical remission was defined as a reduction in Lichtiger’s CAI of ≤4. Basically, we evaluated the efficacy of biologics at approximately 52 weeks of therapy. Additionally, we examined improvements in the C-reactive protein (CRP) levels of patients after biologic therapy.

### 2.3. Assessment of Endoscopic Improvement

Endoscopic improvement was evaluated using the Mayo endoscopic subscore (MES) [41] after biologic therapy. Endoscopic improvement was defined as a subscore of 0 or 1 on the Mayo endoscopic component (originally termed “mucosal healing”).

### 2.4. Statistical Analysis

Statistical analysis was performed using a chi-squared test, Wilcoxon signed-rank test, or Mann–Whitney U test. A *p*-value of ≤0.05 was considered statistically significant.

## 3. Results

### 3.1. Baseline Characteristics of Patients

Of the 81 patients with refractory UC who underwent CAP, 30 were eligible for inclusion in the study (male, *n* = 13, female, *n* = 17; mean age 47.0 years). Of the 30 patients with UC, 16 did not respond to non-response to previous CAP, and 14 had experienced an insufficient response to previous CAP. Detailed clinical characteristics of the patients are presented in Table 1.

### 3.2. Clinical Efficacy of Biologics in Patients with UC Who Underwent CAP Before Biologic Therapy

**Figure 1:**
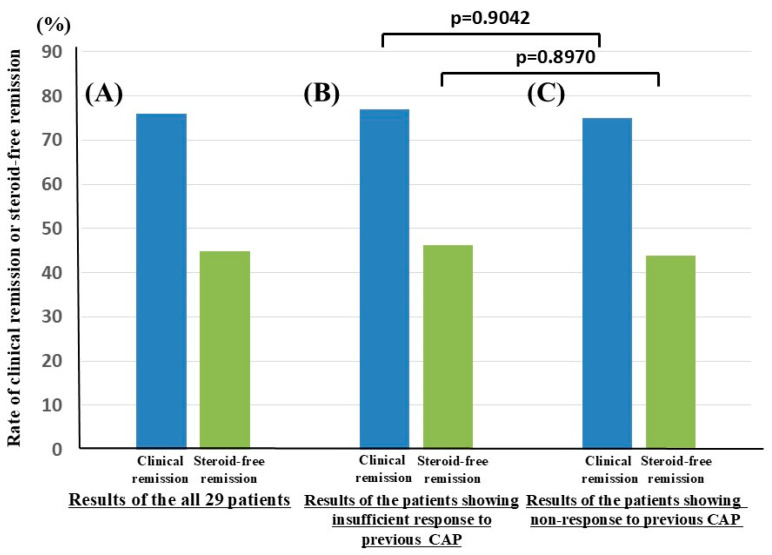
Efficacy of biologics in patients with UC who had undergone CAP before biologic therapy. The rate of clinical or steroid-free remission is shown with a blue or green column, respectively. (**A**) The rates of clinical or steroid-free remission in all 29 patients with UC who had undergone CAP prior biologics are shown. (**B**) The rates of clinical or steroid-free remission in UC patients who experienced an insufficient response to previous CAP are shown. (**C**) The rates of clinical or steroid-free remission in UC patients who did not respond to previous CAP are shown.

One female steroid-dependent UC patient developed persistent cough, which led to the discontinuation of biologic therapy (ADA) within 4 weeks. Therefore, we evaluated the efficacy of biologics in 29 patients with UC. Following the administration of biologic therapy, the clinical and steroid-free remission rates in patients with UC who had undergone CAP prior to biologics were 75.9% and 44.8%, respectively (Figure 1A).

**Figure 2:**
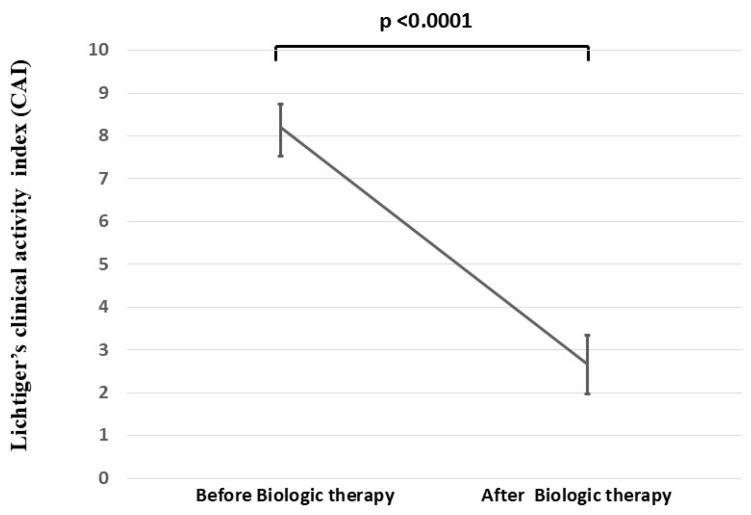
Mean Lichtiger’s CAI scores of the 29 patients with UC before and after biologic therapy. The changes in mean Lichtiger’s CAI scores before and after biologic therapy of the 29 patients with UC are shown.

The mean CAI score (mean ± standard error [SE]) of the patients was significantly reduced from 8.21 ± 0.53 to 2.66 ± 0.68 after biologic therapy (*p* < 0.0001) (Figure 2). 

**Figure 3:**
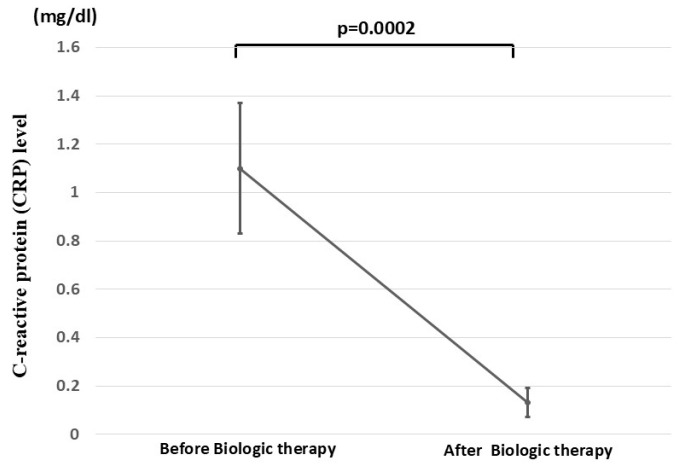
Mean CRP level of the 29 patients with UC before and after biologic therapy. The changes in mean CRP level of the 29 patients with UC before and after biologic therapy are shown.

Additionally, we observed that the mean CRP level (mean ± SE) was significantly reduced from 1.10 ± 0.27 mg/dL to 0.13 ± 0.06 mg/dL following biologic therapy (*p* = 0.0002) (Figure 3).

Among the 29 patients, those who experienced an insufficient response to previous CAP exhibited clinical and steroid-free remission rates of 76.9% and 46.2%, respectively, following biologic therapy (Figure 1B). In patients who did not respond to previous CAP, biologic therapy elicited clinical and steroid-free remission rates of 75.0% and 43.8%, respectively (Figure 1C). Following biologic therapy, there were no significant differences in clinical (*p* = 0.9042) and steroid-free remission rates (*p* = 0.8970) between patients who responded insufficiently to CAP and those who did not respond to CAP before biologic therapy.

The clinical remission rates in the patients treated with IFX, ADA, VDZ, and UST were 9/11 (81.8%), 8/10 (80.0%), 4/7 (57.1%), and 1/1 (100%), respectively. The steroid-free remission rates in patients treated with IFX, ADA, VDZ, and UST were 6/11 (54.5%), 4/10 (40%), 3/7 (42.9%), and 0/1 (0%), respectively.

**Figure 4:**
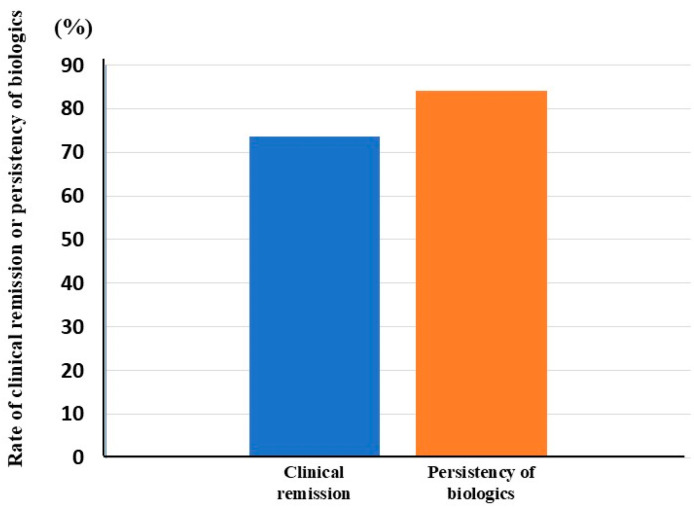
Rates of maintenance of clinical remission and persistency of biologics at 104 weeks of therapy. The rate of clinical remission or persistency of biologics is shown with a blue or orange column, respectively.

We evaluated rates of maintenance of clinical remission and persistency of biologics at 104 weeks of therapy in the patients who had achieved clinical remission at 52 weeks of biologic therapy. The rates of maintenance of clinical remission and persistency of biologics at 104 weeks of therapy were 73.7% and 84.2%, respectively (Figure 4).

**Figure 5:**
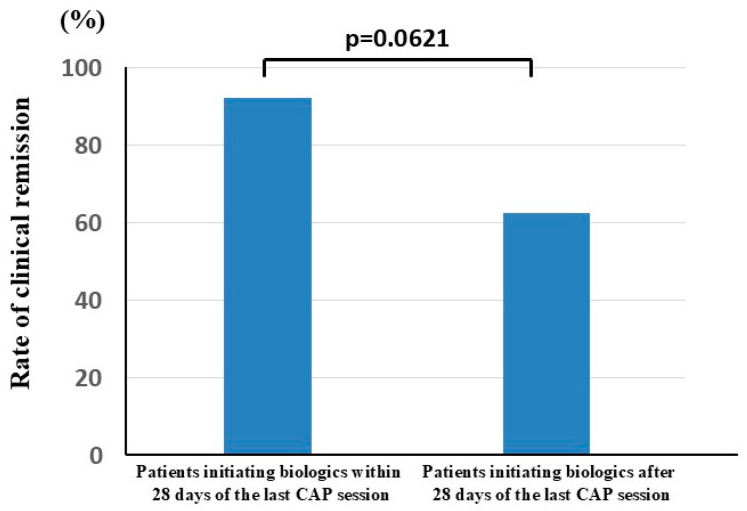
The rates of remission in patients initiating biologics within 28 days of the last CAP session and patients initiating biologics after 28 days of the last CAP. The rate of clinical remission is shown with blue column. The rates of remission in patients initiating biologics within 28 days of the last CAP session (left column) and patients initiating biologics after 28 days of the last CAP session (right column) are shown.

The duration between previous CAP failure and biologic initiation was 1 day to 4015 days (median 56 days) (Table 1). The rates of remission in patients who initiated biologic therapy within 28 days of the last CAP session and the patients who initiated biologic therapy after 28 days of the last CAP session were 92.3% and 62.5%, respectively (Figure 5). The rates of steroid-free remission in the patients who initiated biologic therapy within 28 days of the last CAP session and the patients who initiated biologic therapy after 28 days of the last CAP session were 61.5% and 31.3%, respectively. Although the rates of remission and steroid-free remission tended to be high in patients initiating biologic therapy within 28 days after the last CAP session compared with those in patients initiating biologic therapy after 28 days of the last CAP, significant differences were not observed in clinical remission (*p* = 0.0621) and steroid-free remission (*p* = 0.1029) rates between the two groups.

In addition, there were no significant differences in CAI scores at initiation of biologics (*p* = 0.6434) and disease duration (*p* = 0.6029) between the patients who achieved clinical remission and those who did not respond to biologic therapy.

The mean serum albumin concentration (mean ± SE) at initiation of biologics was 3.66 ± 0.10 g/dL (Table 1). The mean serum albumin concentrations at initiation of biologics in the patients who achieved clinical remission after biologic therapy and the patients who did not respond to biologics were 3.72 ± 0.12 g/dL and 3.47 ± 0.24 g/dL, respectively. Although the mean serum albumin concentration tended to be slightly high in patients who achieved clinical remission compared with that in patients who did not respond to biologics, a significant difference was not observed (*p* = 0.3203) in the mean serum albumin concentration between the two groups.

Of the 29 patients, 17 had steroid-dependent UC patients and 12 had steroid-refractory UC. Following biologic therapy, patients with steroid-dependent UC had clinical and steroid-free remission rates of 82.4% and 41.2%, respectively. Patients with steroid-refractory UC exhibited clinical and steroid-free remission rates of 66.7% and 50%, respectively. Significant differences were not observed in rates of clinical and steroid-free remission between patients with steroid-dependent and steroid-refractory UC treated with biologics.

### 3.3. Endoscopic Improvement After Biologic Therapy

Following biologic therapy, 22 of the 29 patients underwent a colonoscopic examination. Endoscopic improvement (mucosal healing) was observed in 12/22 (54.5%) of the patients.

### 3.4. Adverse Events

During biologic therapy, three patients experienced adverse events (herpes virus infection, *n* = 1; cough, *n* = 1; and skin symptoms, *n* = 1) during biologic therapies. Although no serious adverse events were observed, treatment with biologics was discontinued in one patient owing to a persistent cough.

## 4. Discussion

Our study revealed that biologic therapy was associated with high rates of clinical remission and steroid-free remission, specifically 75.9% and 44.8%, respectively, in patients with refractory UC who had experienced non-response or insufficient response to previous CAP. Furthermore, the biologics examined in this study (IFX, ADA, VDZ, and UST) elicited clinical remission rates exceeding 50%. Overall, 54.5% of patients treated with the biologics exhibited mucosal improvement. In addition, the rates of maintenance of clinical remission and persistency of biologics at 104 weeks of therapy were 73.7% and 84.2%, respectively.

However, we could not compare these results with those of patients with UC who did not experience CAP before biologic therapy, as only three such patients were available in our cohort. Of these three patients, one (33.3%) achieved steroid-free remission, while two (66.7%) achieved clinical remission. Therefore, we compared the results of this study with those of previous studies.

In patients with UC treated with IFX, ADA, VDZ, and UST, these treatments could reportedly elicit clinical remission rates of 38.8–70% [2,42], 16.5–47.8% [2,43,44,45], 16.9–61.9% [21,22,43,44,46,47,48], and 15.5–69.2% [43,49,50,51,52,53], respectively. Additionally, rates of steroid-free remission in patients with UC treated with IFX, ADA, VDZ, and UST were from 39.7 to 61.5 [2], 21.8 to 67.3% [2,44], 12.6 to 45.2% [21,44,46,47,48], and 39 to 67.8% [49,50,52,53], respectively. Moreover, patients with UC were shown to exhibit mucosal improvement rates of 32–62% [2,42], 27.7–42.9% [44,45], 39.7–62.2% [21,22,44,46,47], and 51.1–58.2 [52,53] upon treatment with IFX, ADA, VDZ, and UST, respectively. A history of anti-TNF-α therapy or treatment may negatively impact clinical remission and response to second-line biologics [50,53]. In this regard, the results of previous studies included those of both patients with UC who were exposed to biologics and those who were unexposed to biologics. However, all 30 patients with UC enrolled in our current study were not exposed to biologics. Thus, we conducted a literature search to identify previous studies that examined the efficacy of biologic therapy in biologic-naïve patients with UC and compared the results of the current study more accurately. The efficacy of biologic therapy in biologic-naïve patients with UC as well as biologic-exposed patients with UC, as reported in previous reports, is summarized in Table 2. 

According to Table 2, the clinical remission rates in biologic-naïve patients with UC who received IFX, ADA, VDZ, and UST were found to be 34–48.6% [54,55,56,57], 10–24.3% [43,44,57], 23.0–67.5% [43,44,46,47,54,55,56,57], and 18.4–80% [43,49,50,54], respectively. The rates of steroid-free remission in biologic-naïve patients with UC treated with IFX, ADA, VDZ, and UST were 44.4% [55], 21.7% [44], 14.9–67.5% [44,46,47,55], and 70.9–77% [49,50], respectively. Furthermore, biologic-naïve patients with UC who received IFX, ADA, and VDZ therapy had mucosal improvement rates of 11.8–80.6% [55,56,57,58], 29.5–49% [44,57], and 29.5–86.6% [44,46,47,55,56,57,58], respectively. Next, we compared the results of the current study with those of previous studies. Despite differences in patient characteristics, heterogeneity of the data collected in each study, and differences in methods used to evaluate clinical remission, the rates of clinical remission, steroid-free remission, and mucosal improvement in biologic therapy in our study were not inferior to those of previous studies, including those studies comprising biologic-naïve patients with UC, and were approximately similar to those of earlier studies. Thus, in this study, we demonstrated that biologic therapy was also effective in patients with refractory UC who had experienced non-response or insufficient response to previous CAP, suggesting that biologics may be valuable as a second-line therapy in these patients.

**Table 2:** Efficacy of biologics in biologic naïve and exposed patients with ulcerative colitis.

Biologics [Ref. No. *]		Rate of Clinical Remission (Biologic Naïve and Exposed Patients **)	Rate of Steroid-Free Remission (Biologic Naïve and Exposed Patients)	Rate of Endoscopic Improvement (Biologic Naïve and Exposed Patients)
**Iufliximab**	[54]	36.4%		
	[58]			47.1%
	[55]	44.4%	44.4%	11.8%
	[56]	48.6% ***		80.6% ***
	[57]	39%		60%
	[57]	34%		
**Adalimmab**	[43]	21.3% and 9.2%		
	[57]	18%		47%
	[57]	21%		49%
	[57]	10%		41%
	[44]	24.3% and 16.0%	21.7% and 22.2%	29.5% and 21.0%
**Vedolizumab**	[54]	32.4%		
	[58]			65.6%
	[55]	67.5%	67.5%	29.5%
	[43]	23.1% and 9.8%		
	[57]	23% and 10%		49% and 30%
	[44]	34.2% and 20.3%	14.9% and 4.2%	43.1% and 26.6%
	[46]	46.9% and 36.1%	44.6% and 26.7%	60.0%and 44.6%
	[47]	55.9% and 62.5%	34.6% and 35.7%	61.5% and 63.6%
	[56]	65.9%		86.6%
**Ustekinumab**	[54]	43.3%		
	[43]	18.4% and 12.7%		
	[49]	72.2% and 62.6%	70.9% and 60.4%	
	[50]	80% and 54%	77% and 48%	

Ref. No. *: reference number, **:data from biologic naïve patients are shown in black and from biologic exposed patients are shown in red, ***: data from patients treated with an anti-TNFα (infliximab, adalimumab, golimumab).

In this study, there were no significant differences in CAI scores at initiation of biologics and disease duration between the patients who achieved clinical remission and those who did not respond to biologic therapy. Though no significant differences were observed, the rates of remission and steroid-free remission tended to be higher in patients initiating biologic therapy within 28 days after the last CAP session compared with those in patients initiating biologic therapy after 28 days of the last CAP session. Thus, it may be better to start biologic therapy earlier after the end of CAP therapy. However, it needs to be addressed that this study did not have a control group, and the results were compared with those of previous studies. In addition, there may have been potential selection bias in our study, as a limited number of patients were selected from a single institution. Recent studies have determined the effectiveness of second-line agents in patients with UC who failed to respond to first-line biologic therapy [10,11,12,13,14,15,16]. However, the efficacy of second-line agents in patients with UC who failed to respond to prior CAP treatment remains to be established. Therefore, our results contribute to the optimization of second-line agents for patients with refractory UC who failed first-line CAP treatment.

Recently, additional biologics and small molecules with distinct mechanisms of action were approved for the treatment of UC, providing options for both first-line and subsequent therapies [9,12]. However, there is limited evidence on comparative efficacy, along with considerable gaps in the data regarding drug efficacy when administered in different sequential orders [12]. To the best of our knowledge, no studies have examined the therapeutic sequence of biologics and CAP. Therefore, it is difficult to establish which therapy should be selected as a first-line therapy for patients with refractory UC, specifically biologics or CAP. According to Kapizioni et al., the selection of a first-line therapy would ideally be informed by high-quality evidence drawn from head-to-head short- and long-term comparisons of efficacy, safety, health–economic, and quality-of-life outcomes [12]. We suggest that the following findings are crucial in guiding the selection of a suitable first-line therapy. First, previous studies have suggested that the efficacy of CAP is reduced in patients with UC previously exposed to biologics or in those who experienced failure to biologic therapy [18,19]. Second, our study demonstrated that the efficacy of biologics was not reduced in patients with UC who had experienced a non-response or insufficient response to previous CAP. Although limited, these results suggest that biologics can be useful as a second-line therapy in patients with UC who experience non-response or insufficient response to previous CAP; however, CAP may be unsuitable as a second-line therapy for patients with UC previously exposed to biologics or who experienced failure to biologic therapy.

A known strength of CAP is its excellent safety profile [23,24,25,26,27,32,59]. Hibi et al. [27] evaluated the safety of Adacolumn in 697 patients with UC in 53 medical institutions, revealing the absence of serious adverse events, with only mild to moderate adverse events observed in 7.7% of patients. Conversely, anti-TNF-α therapy may lead to serious infection, demyelinating disease, and associated mortality [19]. Goss Sawney et al. showed that the poor efficacy was the most common reason underlying the discontinuation of biologics for Crohn’s disease and UC, with the occurrence of unwanted side effects considered the second most common reason for the discontinuation of biologics [60]. In patients with both UC and Crohn’s disease, most biologic drugs have been associated with persistent rates of 50–75%+ and 60%+ around one and three years, respectively [60]. Conversely, maintenance therapy with CAP is not essential after the induction of clinical remission with CAP. Re-treatment with CAP was consistently associated with a high rate of effectiveness in patients with UC who had remissive responses during the first course of CAP [29,31]. Although biologics have excellent efficacy in patients with refractory UC, based on the aforementioned findings, we suggest that it may be meaningful to consider CAP before biologic therapy in patients with refractory UC.

Recent studies have demonstrated the efficacy of concomitant use of CAP and biologics in patients with UC who experienced an insufficient response or loss of response to biologics [8]. According to these reports, combination therapies with CAP and biologics safely induced clinical remission or response and steroid-free remission in 32–69% (mean: 47.97%, median: 42.9%) and 9–75% (mean: 40.7%, median: 38%) of patients with UC refractory to biologics, respectively [8]. Rodriguez-Largo et al. suggested that the observed benefits of the combination therapies may be related to multiple mechanisms of action [61]. The authors suggested that GMA could reduce the circulating inflammatory burden in addition to directly improving disease activity and thus allow anti-TNF to restore its response. Hanai et al. [62] showed that the levels of soluble TNF-α receptor I/II significantly increased in the peripheral blood at the end of the GMA session. Furthermore, Yokoyama et al. [63,64] reported that GMA therapy can reduce IFX antibodies and enhance plasma trough levels of IFX in patients with loss of response to IFX. Thus, we suggest that CAP should be added without discontinuation of biologics in patients with UC who showed an insufficient or loss of response to biologic therapy.

## 5. Future Perspectives and Limitations

Our study demonstrated that biologic therapy was effective in patients with refractory UC who had experienced non-response or insufficient response to previous CAP. Nevertheless, the limitations of this study need to be addressed. First, this study was a retrospective study with a small sample size. Second, it lacked a control group with patients with UC who had not undergone CAP before biologic therapy. Third, this study was conducted in a single medical institution. Thus, a multicenter prospective study with a larger sample size is required to validate our findings.

Further, evaluation of the efficacy of biologics in a larger number of patients with an extended follow-up duration would improve the quality of the study.

## 6. Conclusions

To the best of our knowledge, this study is the first to demonstrate that biologic therapy can be effective in patients with refractory UC who experienced a non-response or insufficient response to previous CAP. From the perspective of therapeutic algorithms for biologics and CAP in patients with refractory UC, our results suggest that biologics may be valuable as a second-line therapy for patients with refractory UC with prior failure to first-line CAP therapy. Given prior findings suggesting reduced CAP efficacy in patients with UC after biologic exposure and the excellent safety profile of CAP, it may be meaningful to consider CAP prior to biologic therapy. Nevertheless, additional studies are needed to confirm these findings.

## Figures and Tables

**Table 1:** Baseline characteristics of the patients.

Age (years, mean ± SD)	18–85 (47.0 ± 17.7)	
Sex	Male 13, Female 17	
Disease duration from diagnosis (month, mean ± SD)	10–276 (102.6 ± 81.6)	
Disease extent	Left-sided colitis	8
	Pancolitis	22
Disease refractory type	Steroid-dependent	18
	Steroid-refractory	12
Efficacy of the previous CAP		
Non-response	16	
Insufficient response	14	
Mean CAI * (mean ± SE) at CAP initiation	9.70±0.45	
Duration between CAP failure and biologic initiation	1–4015 days (median 56 days)	
Mean CAI (mean ± SE) at initiation of biologics	8.07 ± 0.52	
Mean serum albumin concentration (mean ± SE) at initiation of biologics	3.66 ± 0.10 g/dL	
Name of the biologics used		
Infliximab	11	
Adalimumab	11	
Vedolizumab	7	
Ustekinumab	1	
Medication (before biologic therapy)		
PSL **	Yes 30, No 0	
5-ASA ***	Yes 28, No 2	
Thiopurines	Yes 19, No 11	

CAI *, Lichtiger’s clinical activity index; PSL **, Prednisolone; 5-ASA ***, 5-Aminosalicylic Acid.

## Data Availability

The data underlying this article will be shared upon reasonable request from the corresponding author.

## Abbreviations

The following abbreviations are used in this manuscript:

UC	Ulcerative colitis
IBD	Inflammatory bowel disease
CS	Corticosteroid
CAP	Cytapheretic
GMA	Granulocyte and monocyte adsorptive apheresis
LCAP	Leukocytapheresis
TNF-α	Tumor necrosis factor-α
CAI	Clinical activity index
IFX	Infliximab
ADA	Adalimumab
VDZ	Vedolizumab
UST	Ustekinumab
